# Adverse effects of adaptive mutation to survive static culture conditions on successful fitness of the rice pathogen *Burkholderia glumae* in a host

**DOI:** 10.1371/journal.pone.0238151

**Published:** 2020-08-24

**Authors:** Gi-Young Kwak, Eunhye Goo, Haeyoon Jeong, Ingyu Hwang

**Affiliations:** 1 Department of Agricultural Biotechnology, Seoul National University, Seoul, Republic of Korea; 2 Research Institute of Agriculture and Life Sciences, Seoul National University, Seoul, Republic of Korea; University of Zurich, SWITZERLAND

## Abstract

Bacteria often possess relatively flexible genome structures and adaptive genetic variants that allow survival in unfavorable growth conditions. Bacterial survival tactics in disadvantageous microenvironments include mutations that are beneficial against threats in their niche. Here, we report that the aerobic rice bacterial pathogen *Burkholderia glumae* BGR1 changes a specific gene for improved survival in static culture conditions. Static culture triggered formation of colony variants with deletions or point mutations in the gene *bspP* (BGLU_RS28885), which putatively encodes a protein that contains PDC2, PAS-9, SpoIIE, and HATPase domains. The null mutant of *bspP* survived longer in static culture conditions and produced a higher level of bis-(3′-5′)-cyclic dimeric guanosine monophosphate than the wild type. Expression of the bacterial cellulose synthase regulator (*bcsB*) gene was upregulated in the mutant, consistent with the observation that the mutant formed pellicles faster than the wild type. Mature pellicle formation was observed in the *bspP* mutant before pellicle formation in wild-type BGR1. However, the population density of the *bspP* null mutant decreased substantially when grown in Luria–Bertani medium with vigorous agitation due to failure of oxalate-mediated detoxification of the alkaline environment. The *bspP* null mutant was less virulent and exhibited less effective colonization of rice plants than the wild type. All phenotypes caused by mutations in *bspP* were recovered to those of the wild type by genetic complementation. Thus, although wild-type *B*. *glumae* BGR1 prolonged viability by spontaneous mutation under static culture conditions, such genetic changes negatively affected colonization in rice plants. These results suggest that adaptive gene sacrifice of *B*. *glumae* to survive unfavorable growth conditions is not always desirable as it can adversely affect adaptability in the host.

## Introduction

Bacterial genomes are flexible and can be easily changed under different growth conditions for survival [1]. Bacteria often acquire beneficial genetic traits from mutations to adapt to their environment [1]. Although bacterial adaptation to variable environmental conditions is essential, it is not always possible to track such changes easily. Thus, experimental evolution of bacteria is useful for the analysis of genetic changes that occur in a relatively short period of time and can improve our understanding of the bacterial response to environmental cues in nature [1–4].

The occurrence of mutations under specific bacterial growth conditions, such as bacterial biofilm microcosms, is considered a competitive and defensive response for survival [3, 5]. Synthesized bacterial cellulosic biofilms float at the air–liquid interface as pellicles where available oxygen is most abundant, and cells with various colony forms exist at the interface [3, 6, 7]. Spatially constrained environments, such as static culture conditions in which sufficient oxygen for normal growth of aerobic bacteria is lacking, require aerobic bacteria to survive and adapt in an oxygen-deficient environment [7–9]. This forces them to evolve in ways that maximize their metabolism for survival [10–12].

Considering that aerobic plant pathogenic bacteria infect plant tissues and proliferate in the intercellular space, it is worth noting the physiological conditions of the apoplast. While it is known that the intercellular space of plant cells is filled with air, the reason for this is unclear [13–16]. The environment of the apoplast, however, is quite different from the artificial shaking culture that supplies sufficient air and nutrients. Therefore, it would be interesting to understand the survival strategies of plant pathogenic bacteria in oxygen-limited conditions in contrast to artificial shaking culture. We used the rice bacterial pathogen *Burkholderia glumae* BGR1 as a model organism and investigated how it genetically and physiologically adapts to oxygen-limited conditions. As an aerobic bacterium, *B*. *glumae* causes rice panicle blight that can result in serious economic damage when weather conditions are favorable for infection and colonization [17, 18]. This bacterium uses amino acids as carbon and nitrogen sources in culture media such as LB [19]. Use of amino acids as carbon sources releases ammonia as a result of deamination, which causes an alkaline environmental pH [20]. High alkalinity is toxic to *B*. *glumae*; the wild-type strain BGR1 produces the strong acidic compound oxalate to detoxify the alkalinity in a quorum sensing (QS) manner [20]. Such detoxification is essential for the survival of *B*. *glumae* when grown in amino acid-rich media [19]. We previously reported that *B*. *glumae* produces cellulase-sensitive pellicles dependent on bis-(3′-5′)-cyclic dimeric guanosine monophosphate (c-di-GMP) but independent of QS in static culture [21], which led us to investigate how cells of *B*. *glumae* behave physiologically and genetically in a pellicle-forming environment.

In this study, we addressed three specific questions regarding the survival and adaptation of *B*. *glumae* in static culture conditions. First, we determined survival behaviors of *B*. *glumae* in static culture compared to shaking culture. Second, we determined whether *B*. *glumae* undergoes genetic changes to improve survival in static culture. Finally, we characterized colony variants that increased survival rates in static culture. We found that static culture conditions triggered genetic changes in the gene *bspP* (BGLU_RS28885), possibly encoding a protein of 108 kDa. Such mutants showed altered colony morphologies that differed from that of the smooth wild-type colony, formed pellicles faster, and exhibited improved survival compared to the wild type in static culture but failed to colonize successfully in the host. Our results suggest that adaptive evolution by sacrifice of a specific gene to overcome unfavorable growth conditions could cause unexpected and devastating outcomes for plant pathogenic bacteria such as *B*. *glumae*.

## Materials and methods

### Bacterial strains and growth conditions

The bacterial strains and plasmids used in this study are listed in S1 Table in S1 File. *B*. *glumae* and *Escherichia coli* were aerobically grown at 250 rpm and 37°C in LB broth (0.1% tryptone, 0.5% yeast extract, and 0.5% NaCl; USB Corp.) with the following antibiotics: ampicillin, 100 μg/mL; kanamycin, 50 μg/mL; tetracycline, 10 μg/mL; trimethoprim, 75 μg/mL; rifampicin, 100 μg/mL; and spectinomycin, 100 μg/mL. LB agar medium contains 1.5% (w/v) agar (BD Biosciences). *B*. *glumae* strains were grown at 28°C and 37°C in 2 ml LB broth in 24-well plates (Corning, Inc.) as static culture. Aliquots of 100 μl from 24-well plates were serially diluted and spread on LB agar medium on day 0 through 7. The harvested samples were taken from different wells of 24- well plate to minimize possible interference involved in daily sampling for monitoring colony forming unit (CFU) colony morphology. Hence, the 7 wells were inoculated with the same sample for each day’s sampling for a week. The plates were incubated at 37°C for 24 h to allow colonies to develop and at room temperature up to 2 days. Colony variants were isolated on day 3 of incubation at 28°C.

### Pellicle assays

Cells were inoculated in 2 mL LB broth with appropriate antibiotics and grown at 37°C at 250 rpm for 18 h. Overnight cultures were washed twice with fresh LB broth, and turbidity was adjusted to an optical density of 0.05 at 600 nm using an Eppendorf BioSpectrometer kinetic (Eppendorf) followed by 2 ml subculture in each of 24-well plates (Corning, Inc.). Pellicles were formed and harvested from the 3^rd^ day of incubation period.

### Nucleic acid extraction and manipulation

Standard DNA cloning, restriction mapping, and gel electrophoresis methods were used [22]. Vector plasmid DNA and genomic DNA were treated with appropriate restriction enzymes as recommended by the suppliers (New England Biolabs and TaKaRa). Extraction of DNA fragments from agarose gels was performed as described by the manufacturer (Qiagen). A previously constructed cosmid genomic library was used [17]. The pLAFR3 and pLAFR6 derivatives were mobilized into *B*. *glumae* strains by triparental mating [23]. Plasmids were extracted with the QIAGEN^®^ Plasmid Mini Kit (Qiagen) according to the manufacturer’s instructions. For purification of PCR products, agarose gel and vector DNA, QIAEX II^®^ Gel Extraction Kit (Qiagen), and BIONEER *AccuPrep*^®^ PCR Purification Kit (Bioneer) were used according to the manufacturers’ instructions. The target gene was amplified via PCR using a thermocycler (Model C1000; Bio-Rad Laboratories) with Phusion DNA polymerase (New England Biolabs).

### Mutagenesis of *bspP*

The mutagenized plasmid carrying a Tn*3*-*gusA* insertion was introduced into the parent strain by bacterial conjugation followed by marker exchange as previously described [24]. The insertion site and orientation of Tn*3*-*gusA* in the mutant were determined by restriction enzyme digestion and direct sequencing of the plasmid using the primer Tn3-gusA (5’-CCGGTCATCTGAGACCATTAAAAGA-3’) as previously described [17]. EZ-Tn*5* insertional mutagenesis of pPAS1 for genetic complementation of the *bspP* null mutant was carried out using the EZ-Tn*5*^TM^ <DHFR-1> Tnp Transposome^TM^ Kit (Epicentre Technologies, Corp.) according to the manufacturer’s instructions. The insertion site was determined using the primers provided in the kit. Marker exchange was confirmed by Southern hybridization analyses.

Assembly and analysis of the CS-type 3-2A mutant strain were performed by preparing and sequencing Illumina shotgun libraries from gDNA in one lane of an Illumina HiSeq 2500 sequencer. Resequenced Illumina HiSeq data were trimmed using Trimmomatic software and aligned to the *B*. *glumae* BGR1 genome using the BWA package. DNA variants in the libraries, including single nucleotide polymorphisms, insertions, and deletions, were detected using SAMtools and FreeBayes [20].

### Oxalate and QS signal assays

*B*. *glumae* was grown in LB or LB supplemented with 100 mM 4-(2-hydroxyethyl) piperazin-1 ethanesulfonic acid (HEPES) to assess oxalate production and QS signals. An oxalate assay kit (Libios) was used according to the manufacturer’s instructions and as described previously [19, 25]. An autoinducer assay was performed as described previously [17] with the following modification: wild-type BGR1 and *bspP* null mutant BGP38 were grown in LB supplemented with HEPES (pH 7.0) for 1 day.

### Quantification of pellicles by cellulase treatment

The harvested 3- to 4-day-old pellicles were washed with Dulbecco’s phosphate-buffered saline (Welgene) followed by treatment with 0.86 units (U)/mL 1,4-(1,3:1,4)-β-D-glucan 4-glucanohydrolase (v/v) (Calbiochem-Novabiochem), 0.1% proteinase K (v/v) (Sigma-Aldrich), or 0.1% RNase-free DNase I (v/v) (Qiagen) as described previously [26, 27]. After overnight incubation at 37°C, the turbidity of the degraded pellicles was measured as the optical density at 600 nm (OD_600_) using an Eppendorf BioSpectrometer kinetic.

### Analyses of c-di-GMP

The extraction of c-di-GMP was performed as described previously [28], and quantification of extracted c-di-GMP was carried out by high performance liquid chromatography (Dionex). Commercially available c-di-GMP (Sigma-Aldrich) was used as a reference.

### Quantitative real-time polymerase chain reaction (qRT-PCR)

Isolation of total RNA from wild-type BGR1, *bspP* null mutant BGP38, and the *bspP* complementation strain BGP38C was performed using an RNeasy Mini Kit (Qiagen) according to the manufacturer’s instructions. DNaseI treatment of the isolated RNA, reverse transcription to cDNA, and RT-PCR were performed as described previously [29]. qRT-PCR was performed as follows: 95°C for 2 min, 30 cycles of 94°C for 30 s, 60°C for 30 s, and 72°C for 30 s, and 72°C for 5 min using a thermocycler (Model C1000; Bio-Rad Laboratories). The primers used for qRT-PCR are listed in S2 Table in S1 File.

### Statistical analysis

Results from the oxalate assay, cellulase assay, and c-di-GMP analysis were compared using analysis of variance with Tukey correction for multiple comparisons. A *P-*value <0.05 was taken to denote statistical significance. The normalized fold expression was calculated for *bcsB* and *obcA*. All analyses were performed on data from three biological replicates using IBM SPSS Statistics software (version 25.0; IBM Corporation).

### Plant inoculation

Virulence and colonization assays of *B*. *glumae* strains in *Oryza sativa* cv. Milyang 23 were performed as described previously [21].

## Results

### Emergence of morphologically distinct spontaneous mutants in static culture

**Fig 1:**
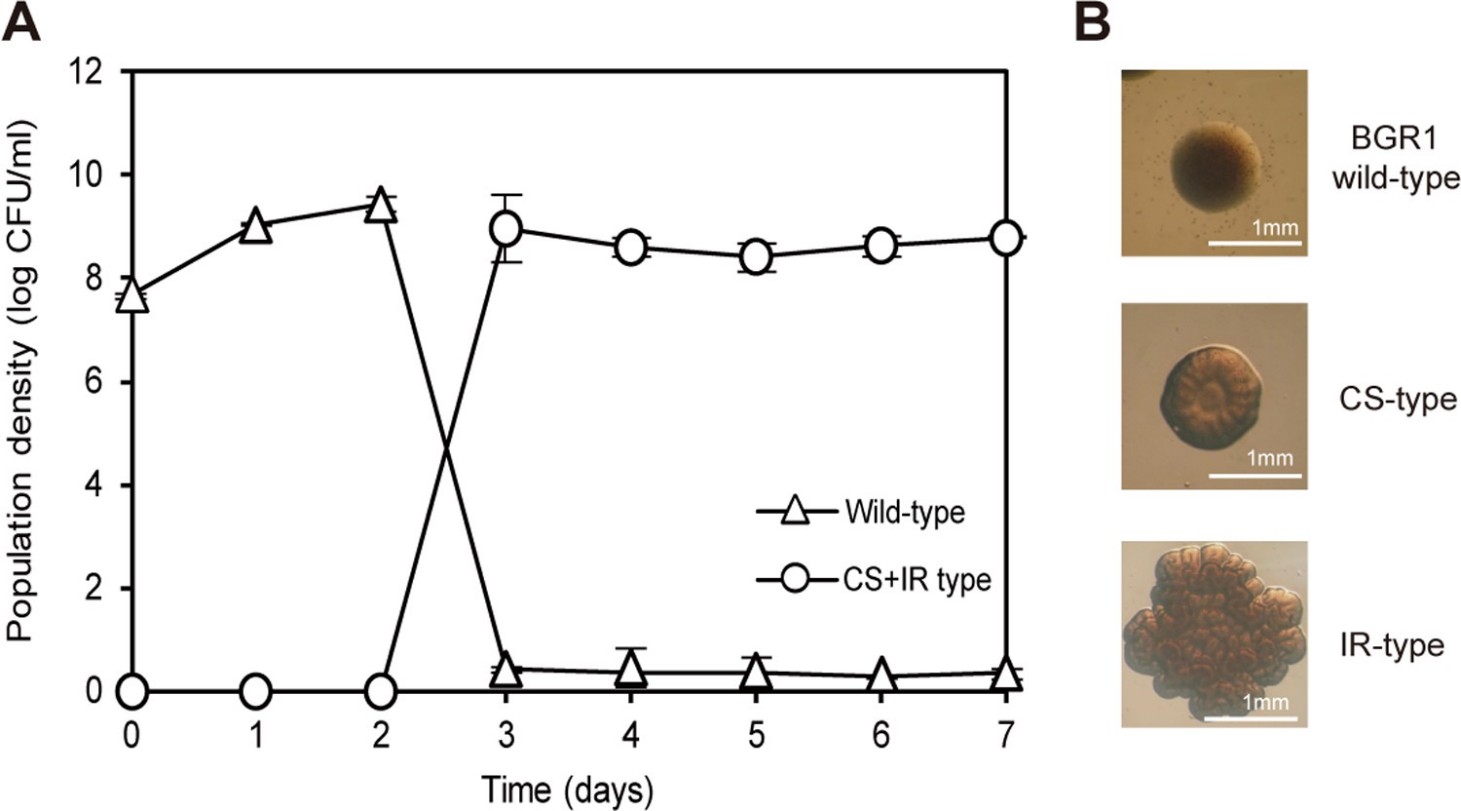
Spontaneously occurring *B*. *glumae* colony variants were produced in static culture. (A) Population density of colony variants was greater than the population density of wild-type BGR1 at day 3 of incubation in static culture at 28°C. Batch cultures of BGR1 prepared strictly from the static culture from day 0 to 7 at 28°C showed an exponential increase in the population density of colony variants. (B) The evolved colony variants had morphologically different shapes compared to wild-type BGR1: colonies with a crater in the middle (CS-type) and colonies with an irregular boundary (IR-type).

To determine the viability of aerobic *B*. *glumae* wild-type BGR1 in static culture, aliquots of the static culture were taken each day for a week. Cell densities increased and reached 1 × 10^9^ CFU/mL 2 days after subculture in 24-well plates (Fig 1A). However, two types of altered colony variants distinctively different from the wild-type BGR1 colonies appeared 3 days after incubation; one had a crater-like shape in the middle of the colony (CS-type) and the other had an irregular boundary (IR-type) (Fig 1B). Combined numbers of both types of colony variants continued to increase exponentially, reaching approximately 90% of whole live cells while the number of total viable cells was maintained at approximately 1 × 10^9^ CFU after 7 days (Fig 1A). On days 3–7 of the static culture incubation period, CS-type colonies dominated the IR-type colonies, occupying 94%, 87%, 80%, 86%, and 88% of the available area, respectively.

### Identification of *bspP* mutations in spontaneous mutants

**Fig 2:**
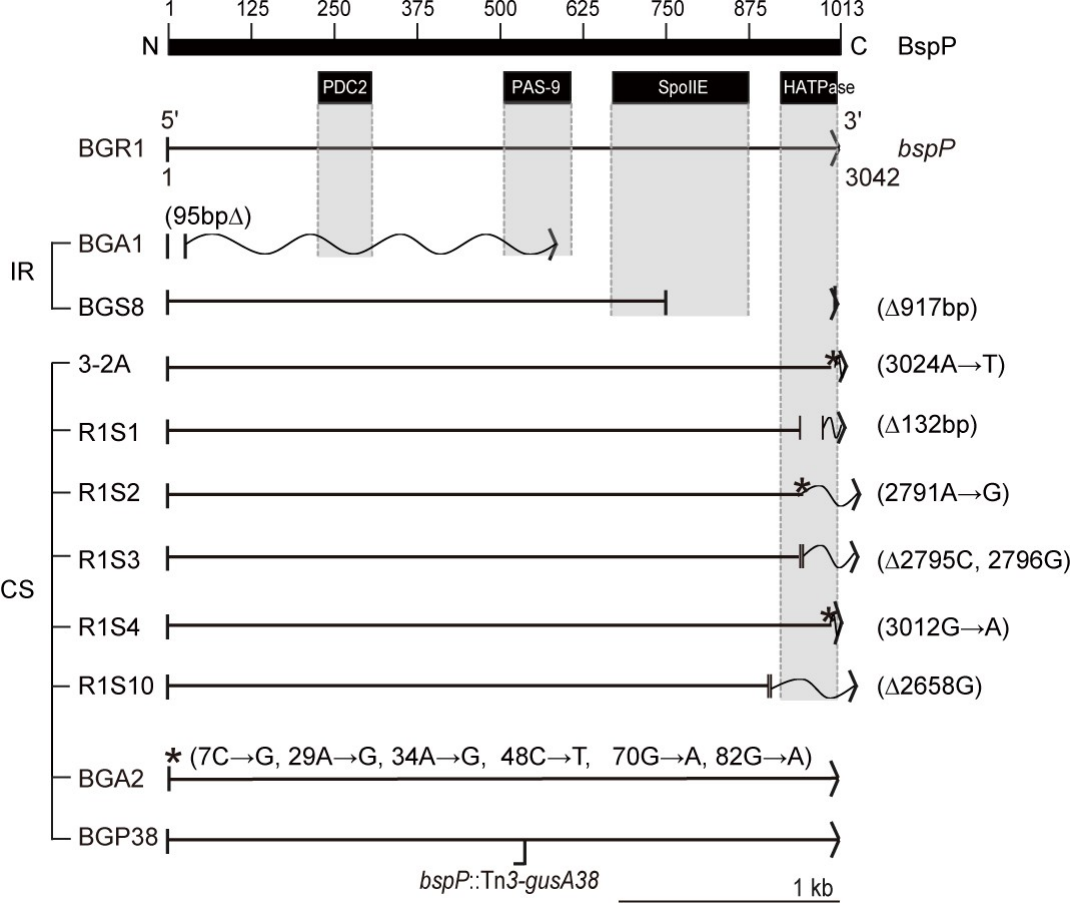
Colony variants in static culture had various mutations in *bspP*. The *bspP* gene consists of 3024 base pairs and putatively encodes 1013 amino acids. Spontaneously evolved IR-type BGA1 and BGS8 had a 95 bp deletion including a putative translation start codon GTG at the 5ʹ end of *bspP* and a 917 bp deletion at the 3ʹ end of *bspP*. Additional spontaneously evolved CS-type colonies in static culture had mutations in *bspP* as follows: strains 3–2A, R1S2, and R1S4 had a point mutation at the 3ʹ end; and strains R1S1, R1S3, and R1S10 had 132, 2, and 1 bp deletions at the 3ʹ end, respectively. The spontaneously evolved CS-type BGA2 had six point mutations all clustered at the 5ʹ end of *bspP*. The CS-type BGP38 (*bspP*::Tn*3*-*gusA38*) represents a Tn*3-gusA* insertional mutation in *bspP*. A solid black bar represents BspP with the conserved domains PDC2, PAS-9, SpoIIE, and HATPase in small solid black boxes below BspP, a thin arrow below BspP represents the *bspP* gene (3042 bp), | | represents deletion mutation regions, *represents point mutation sites, Δ in parentheses represents the number of nucleotides deleted, → in parentheses represents nucleotide changes, vertical dotted lines with grey shades indicate the positions of domains in BspP, and horizontal waves represent faulty proteins after premature termination. Translation termination is denoted with an arrow (>).

To determine whether genetic mutations caused colony morphological changes, we sequenced the whole genome of the CS-type 3–2A mutant. We identified a point mutation at the 3ʹ end of the annotated gene (BGLU_RS28885) called *bspP* (Fig 2). The *bspP* gene was 3042 bp and putatively encodes a protein of 108 kDa with four known domains: second PDC (Pho/Dus/CitA) sensor domain of diguanylate-cyclase (PDC2), signal transduction mechanism domain (PAS-9), stage II sporulation protein E (SpoIIE) domain, and histidine kinase-like ATPase (HATPase) domain (Fig 2).

**Fig 3:**
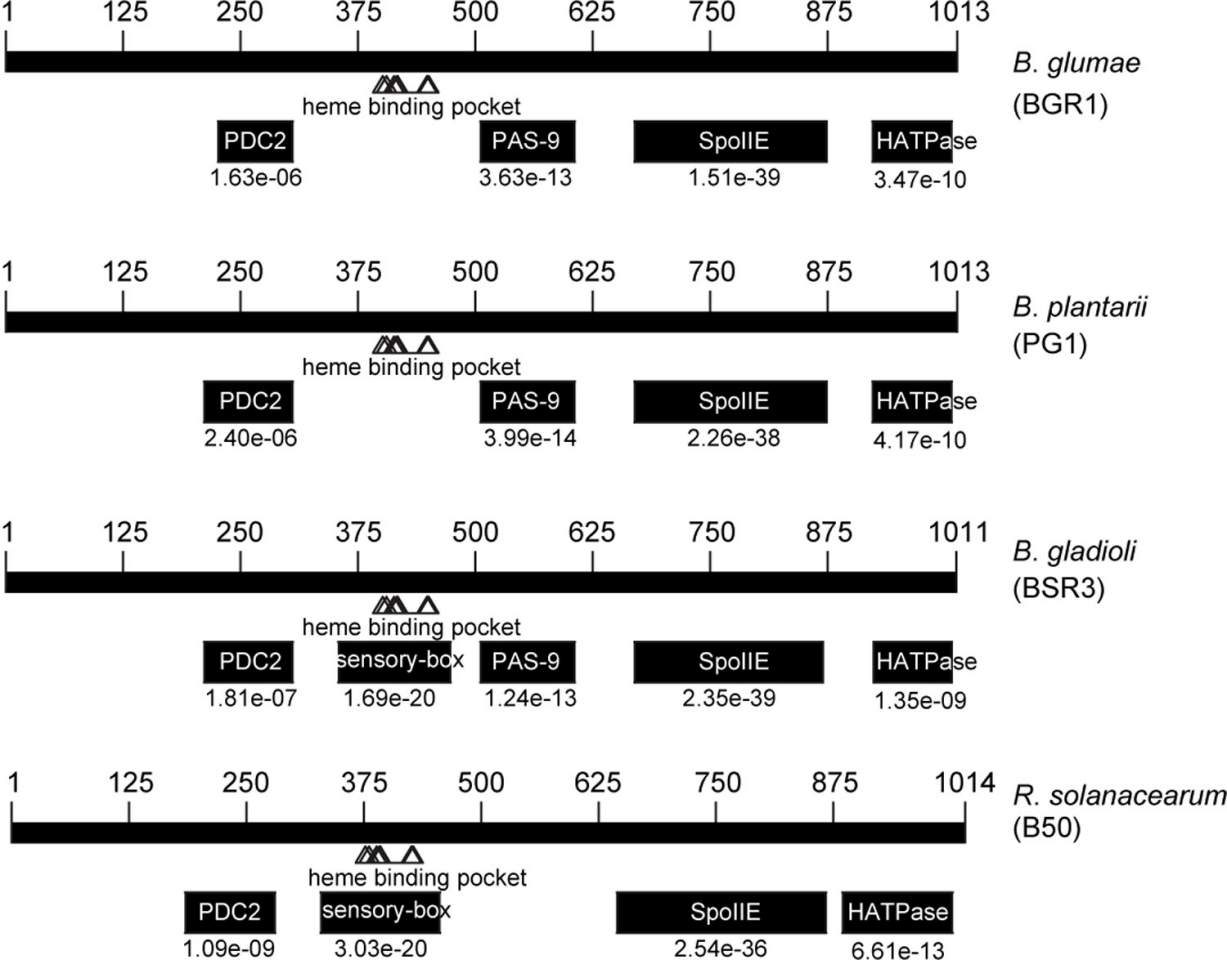
Conserved domains of BspP in *B*. *glumae* and other plant pathogenic bacteria. The following homologous domains were identified in *B*. *glumae*, *B*. *plantarii*, *B*. *gladioli*, and *R*. *solanacearum* with a heme binding pocket: PDC2, SpoIIE, and HATPase. A sensory box was present in *B*. *gladioli* and *R*. *solanacearum*, and no PAS-9 domain was present in *R*. *solanacearum*. The e-values and accession numbers below the representing domains were obtained from GenBank. The respective accession numbers of BspP for *B*. *glumae*, *B*. *plantarii*, *B*. *gladioli*, and *R*. *solanacearum* are as follows: WP_015878232.1, WP_042629168.1, WP_124096127.1, and WP_080894246.1. The identifiable domain accession numbers of homologous BspP in the four species of interest are as follows: PDC2 (cd12915), PAS-9 (pfam13426), SpoIIE (pfam07228), HATPase (cd16936), sensory-box (TIGR00229).

In the 3–2A mutant, nucleotide 3024 (A) in *bspP* was changed to T (denoted as 3024A→T), changing isoleucine to phenylalanine (denoted as I984F) in BspP (Fig 2). To determine whether similar mutations found in the 3–2A mutant occurred in *bspP* in other mutants, we amplified the *bspP* gene in other mutants with the primers BGLU_RS28885F and BGLU_RS28885R (S2 Table in S1 File) followed by direct sequencing of the coding region of *bspP*. We identified various mutations in *bspP* in the spontaneously occurring mutants BGA1, BGS8, R1S1, R1S2, R1S3, R1S4, R1S10, and BGA2 (Fig 2). There was a 95 bp deletion at the 5ʹ end of *bspP* in BGA1, and 132 bp and 917 bp deletions at the 3ʹ ends of *bspP* in R1S1 and BGS8, respectively (Fig 2). One and two base pair deletions were found near the 3ʹ ends of *bspP* in R1S10 and R1S3, respectively (Fig 2). Single point mutations, such as 2791A→G (N926S) and 3012G→A (G980S), were found in R1S2 and R1S4, respectively (Fig 2). Several point mutations (7C→G, 29A→G, 34A→G, 48C→T, 70G→A, and 82G→A) were identified in BGA2, causing the following amino acid changes, respectively: R3G, H10R, T12A, A17A (no amino acid changed due to 48C→T), V24M, and G28R (Fig 2). 3–2A, R1S1, R1S2, R1S3, R1S4, and R1S10 exhibited mutations in the HATPase domain, and BGS8 in the SpoIIE domain of BspP (Fig 2). As a result of the mutations in *bspP*, BGA1 and BGS8 underwent premature termination (Fig 2). To confirm that a mutation in *bspP* confers morphological changes in *B*. *glumae*, we mutagenized pPAS1 carrying *bspP* with Tn*3*-*gusA* followed by marker exchange into wild-type BGR1. The resulting mutant BGP38 (BGR1 *bspP*::Tn*3*-*gusA38*) showed CS-type colony morphology as observed in 3–2A, R1S1, R1S2, R1S3, R1S4, R1S10, and BGA2 (Fig 2). Two other plant pathogenic *Burkholderia* species, *B*. *plantari* and *B*. *gladioli*, possess a homolog of BspP with 94.08% and 84.42% identity at the amino acid level, respectively. Another plant pathogen, *Ralstonia solanacearum*, had a BspP homolog with 51.74% homology (Fig 3). Homologous PDC2, SpoIIE, and HATPas domains were also found in *B*. *plantari*, *B*. *gladioli*, and *R*. *solanacearum*. Heme binding pockets were also present in all four strains with the exception that the sensory-box was present only in in *B*. *gladioli* and *R*. *solanacearum* (Fig 3). The PAS-9 domain was not found in *R*. *solanacearum* (Fig 3). The following accession numbers for *B*. *glumae*, *B*. *plantarii*, *B*. *gladioli*, and *R*. *solanacearum* were obtained from a BLAST search of the NCBI database: WP_015878232.1, WP_042629168.1, WP_124096127.1, and WP_080894246.1, respectively.

### Survival of the *bspP* mutant in static and shaking culture conditions

**Fig 4:**
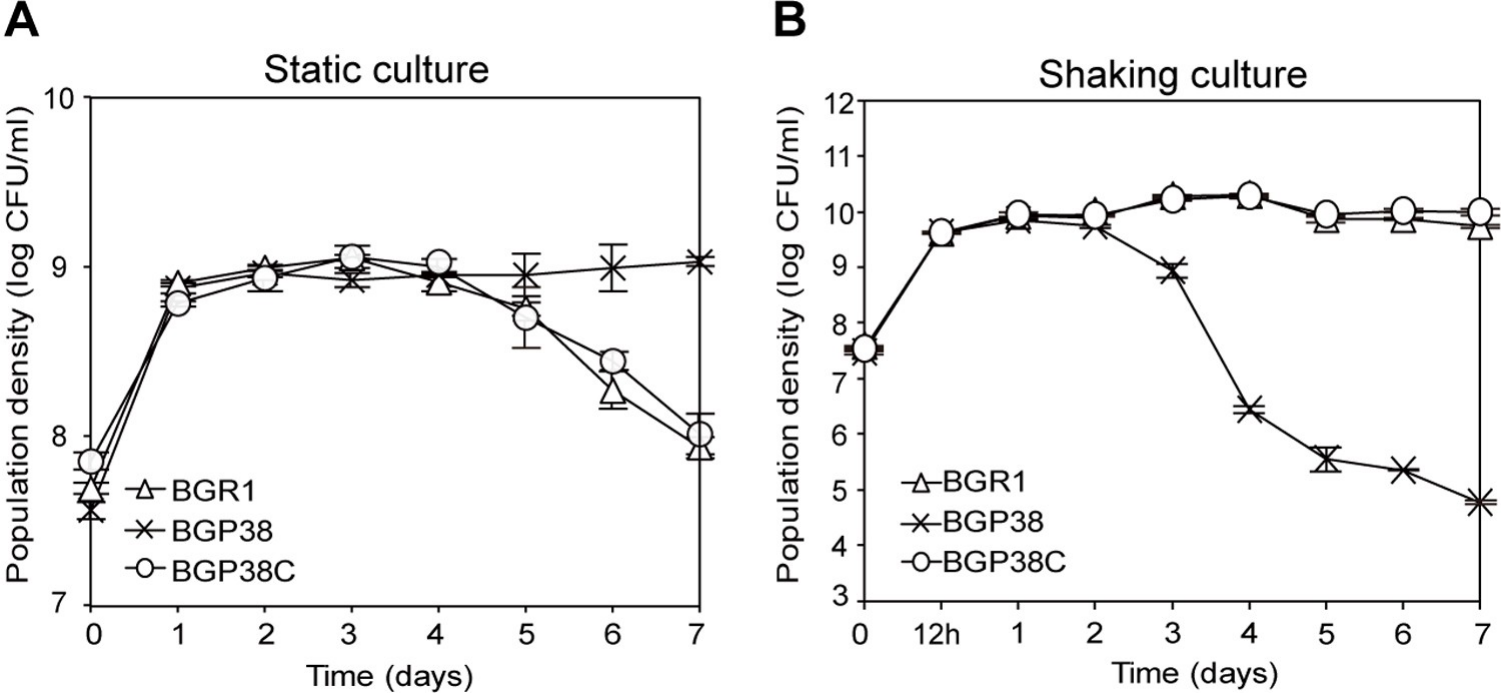
Survival rates of the *bspP* null mutant BGP38 in static and shaking culture. (A) The population density of wild-type BGR1 and the *bspP* complementation strain BGP38C subsided at day 5 after subculture while the *bspP* null mutant BGP38 (*bspP*::Tn*3*-*gusA38*) gradually increased until day 7 of incubation at 28°C. (B) Population density of the *bspP* null mutant BGP38 dropped steeply at day 3 of incubation whereas wild-type BGR1 and the *bspP* complementation strain BGP38C exhibited normal growth without fluctuation.

To determine whether the mutation in *bspP* provides advantageous adaptive tactics for survival in experimental conditions, we determined the viability of the wild-type strain BGR1, the *bspP* null mutant BGP38, and the *bspP* complementation strain BGP38C at 28°C for 1 week in static culture. Densities of all three strains in static culture increased 3–4 days after incubation, and the BGP38 population was maintained at approximately 1 × 10^9^ CFU/mL up to 7 days (Fig 4A). However, the population of wild-type BGR1 and the *bspP* complementation strain BGP38C gradually decreased after 4 days of incubation (Fig 4A). In vigorous shaking culture, wild-type BGR1 and the *bspP* complementation strain BGP38C exhibited normal growth throughout the incubation period; however, population of BGP38 decreased steeply 2 days after inoculation (Fig 4B). This indicated that survival of the *bspP* mutant was dependent upon culture conditions, particularly oxygen availability.

### Physiological characteristics of the *bspP* mutants

**Fig 5:**
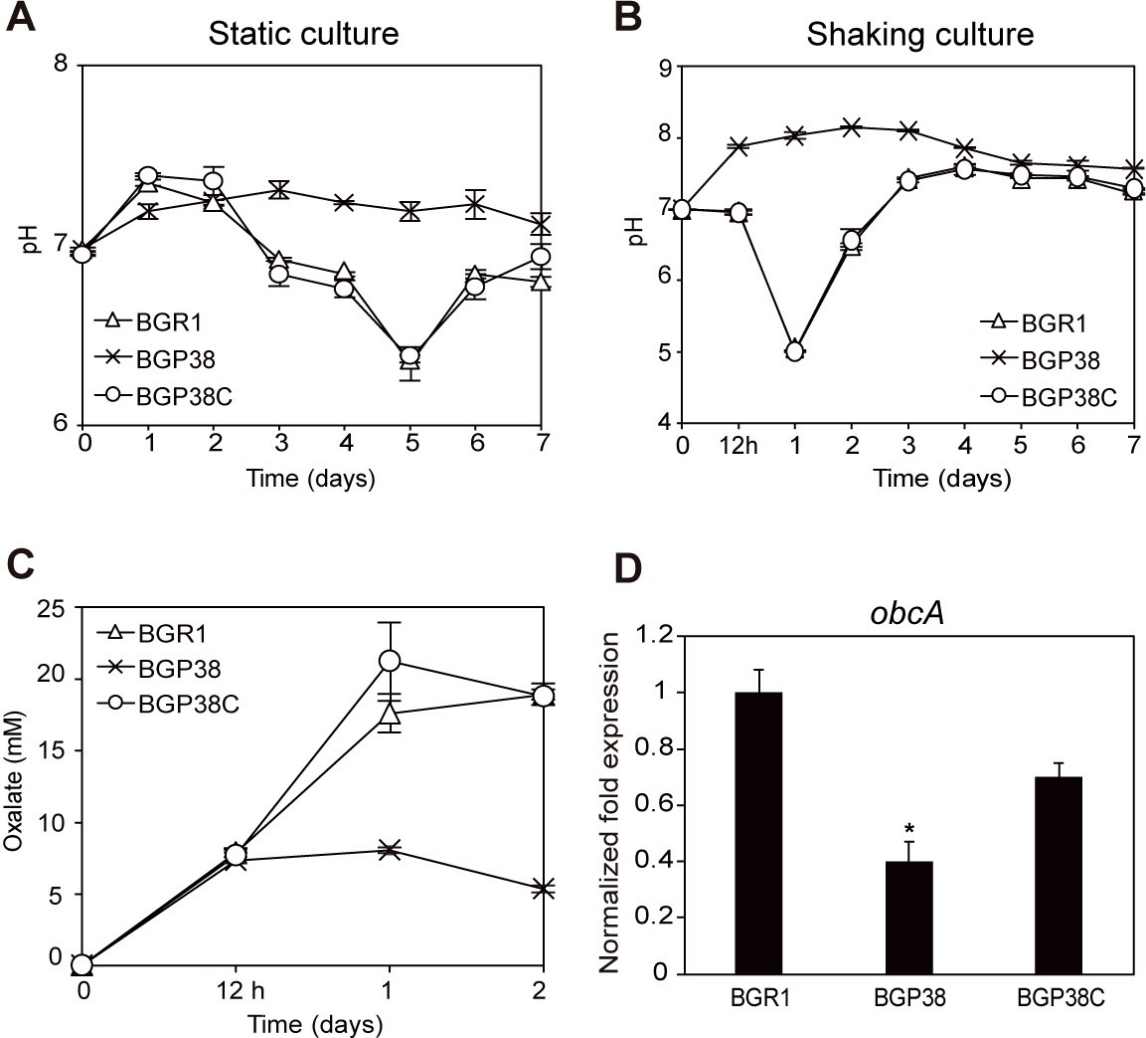
Measurements of environmental pH, oxalate levels, and *obcA* expression in the *bspP* null mutant BGP38. (A) The environmental pH of wild-type BGR1 and the *bspP* complemented strain BGP38C dropped close to 6 at day 5 while the environmental pH of *bspP* null mutant BGP38 was stable at a neutral level from day 0 to 7 in static culture at 28°C. (B) The environmental pH of BGR1 and BGP38C showed normal fluctuation patterns as previously shown in shaking culture whereas the environmental pH of the *bspP* null mutant BGP38 suffered was alkaline. (C) The *bspP* null mutant BGP38 produced less oxalate compared to the levels produced in wild-type BGR1 and the *bspP* complementation strain BGP38C. (D) Expression of *obcA* in the *bspP* null mutant BGP38 was significantly lower than that in wild-type BGR1. The asterisk (*) represents a significant difference (*P* <0.05) in the normalized fold expression of *obcA* among wild-type strain BGR1, BGP38, and BGP38C.

To understand how mutations in *bspP* confer improved survival in static culture compared to the wild type, we monitored changes in the pH of the static culture every day for 7 days. Under static culture conditions, the environmental pH of the *bspP* null mutant BGP38 remained stable at a neutral pH for 7 days (Fig 5A). However, after 2 days in shaking culture, the environmental pH of BGP38 increased to 8.15 (Fig 5B). The environmental pH of BGR1 and the *bspP* complementation strain BGP38C dropped to approximately 5 and recovered to a neutral pH level in both static and shaking culture conditions (Fig 5B).

To understand how the environmental pH of BGP38 was affected by culture conditions, we measured oxalate levels produced by BGP38 grown in LB (Fig 5C) or LB supplemented with HEPES to keep the environmental pH neutral (S1 Fig in S1 File) with shaking for 2 days. We found that BGP38 produced significantly less oxalate than wild-type BGR1 and the *bspP* complementation strain BGP38C in shaking culture with LB (Fig 5C). To determine if there was a correlation between the production of oxalate and expression of the oxalate biosynthetic gene *obcA*, we estimated expression levels of *obcA* in wild-type BGR1, the *bspP* null mutant BGP38, and the *bspP* complementation strain BGP38C. The expression level of *obcA* in the *bspP* null mutant BGP38 was 2.5-fold less than those in wild-type BGR1 and the *bspP* complementation strain BGP38C when grown in LB (Fig 5D). The *bspP* null mutant BGP38 produced both octanoyl- and hexanoyl-L-homoserine lactones to the same levels as those produced by wild-type BGR1 grown in LB supplemented with HEPES (S1 Fig in S1 File), which ruled out the possibility that the decreased oxalate production was due to a lack of QS signal production. The phenotypes of the IR-type *bspP* mutant BGS8 were complemented, as shown in S2 Fig in S1 File.

### Facilitated pellicle formation and upregulation of *bcsB* expression in the *bspP* null mutant BGP38

**Fig 6:**
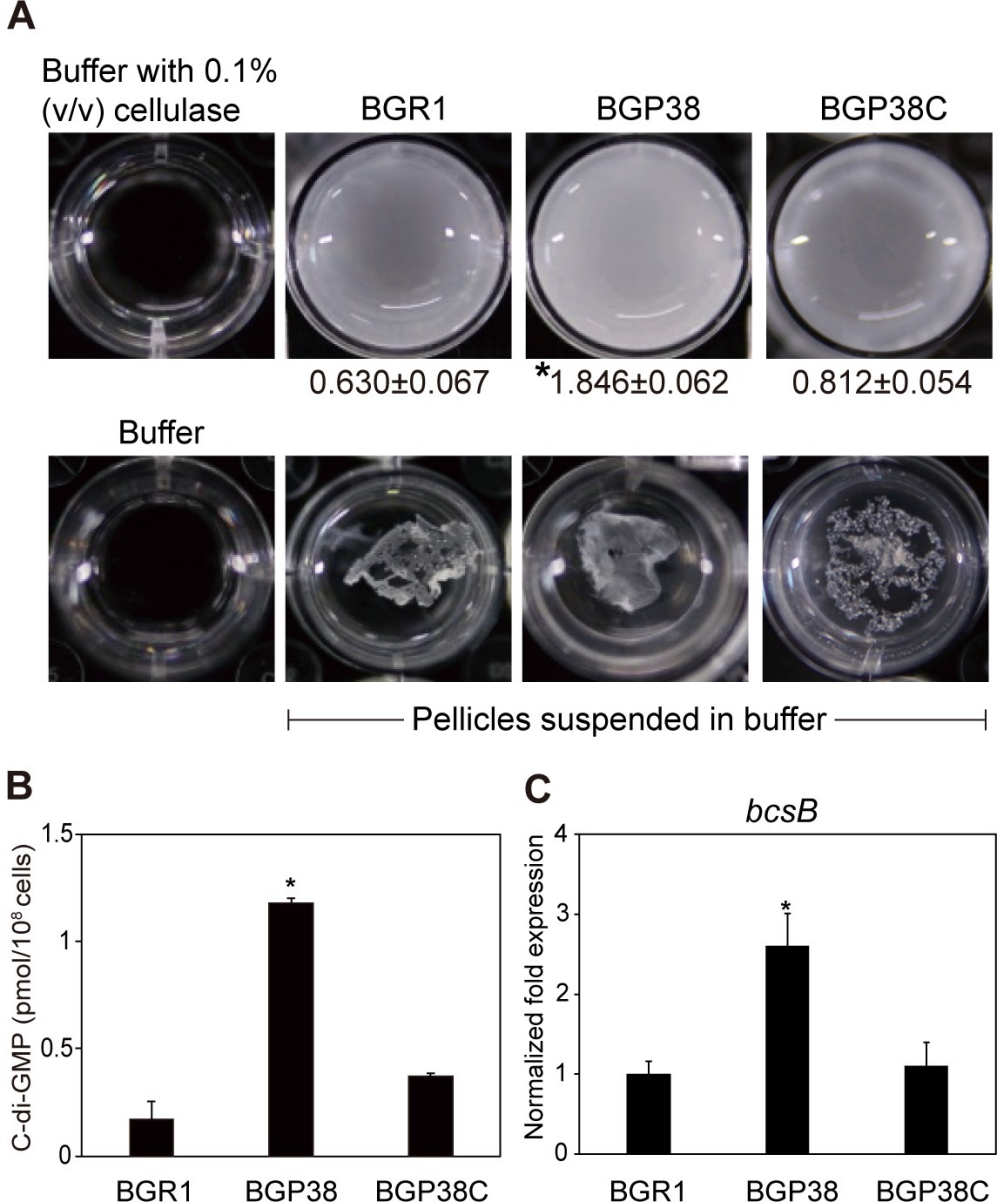
Increased levels of pellicle and c-di-GMP biosynthesis and upregulation of *bcsB* in the *bspP* null mutant BGP38. (A) The levels of cellulase-sensitive pellicles were estimated by turbidity following treatment with 0.1% (v/v) cellulase after 3 days of incubation (mean ± standard deviation). (B) The levels of c-di-GMP were elevated in the *bspP* null mutant BGP38 compared to those of wild-type BGR1 and the complementation strain BGP38C. (C) Expression of the bacterial cellulose synthase regulator gene (*bcsB*, BGLU_RS28235) was significantly higher in the *bspP* null mutant BGP38 than in wild-type BGR1 and the *bspP* complementation strain BGP38C. The asterisks (*) represent significant differences (*P* <0.05) in turbidity after cellulase treatment, c-di-GMP (pmol/10^8^ cells), and normalized fold expression of *bcsB* among wild-type strain BGR1, BGP38, and BGP38C.

In addition to differences in the environmental pH of the *bspP* null mutant BGP38 in static and shaking culture conditions, the key phenotypic difference between wild-type BGR1 and *bspP* null mutant BGP38 was the ability to produce pellicles in static culture (Fig 6A). We measured the turbidity of cellulase-sensitive pellicles produced by wild-type BGR1, the *bspP* null mutant BGP38, and the *bspP* complementation strain BGP38C after cellulase treatment. Turbidities of cellulase-treated pellicles of BGR1, BGP38, and BGP38C were 0.630 ± 0.067, 1.846 ± 0.062, and 0.812 ± 0.054 (mean ± standard deviation), respectively (Fig 6A). These results indicate that the *bspP* null mutant BGP38 produced more cellulase-sensitive pellicles than wild-type BGR1 and the *bspP* complementation strain BGP38C. To confirm that the facilitated pellicle formation in BGP38 was affected by c-di-GMP levels, we measured the levels of c-di-GMP levels extracted from wild-type BGR1 and the *bspP* complementation strain BGP38C. Levels of c-di-GMP in BGP38 were significantly higher than those of wild-type BGR1 and the *bspP* complementation strain BGP38C (Fig 6B). We chose one of the genes involved in cellulose biosynthesis in *B*. *glumae*, *bcsB* (BGLU_RS28235), which encodes a putative cellulose synthase regulator, and assessed its expression in the *bspP* null mutant BGP38. Expression of *bcsB* was significantly higher (2.3-fold) in BGP38 compared to the wild-type strain BGR1 and the *bspP* complementation strain BGP38C (Fig 6C).

### The *bspP* mutant was avirulent

**Fig 7:**
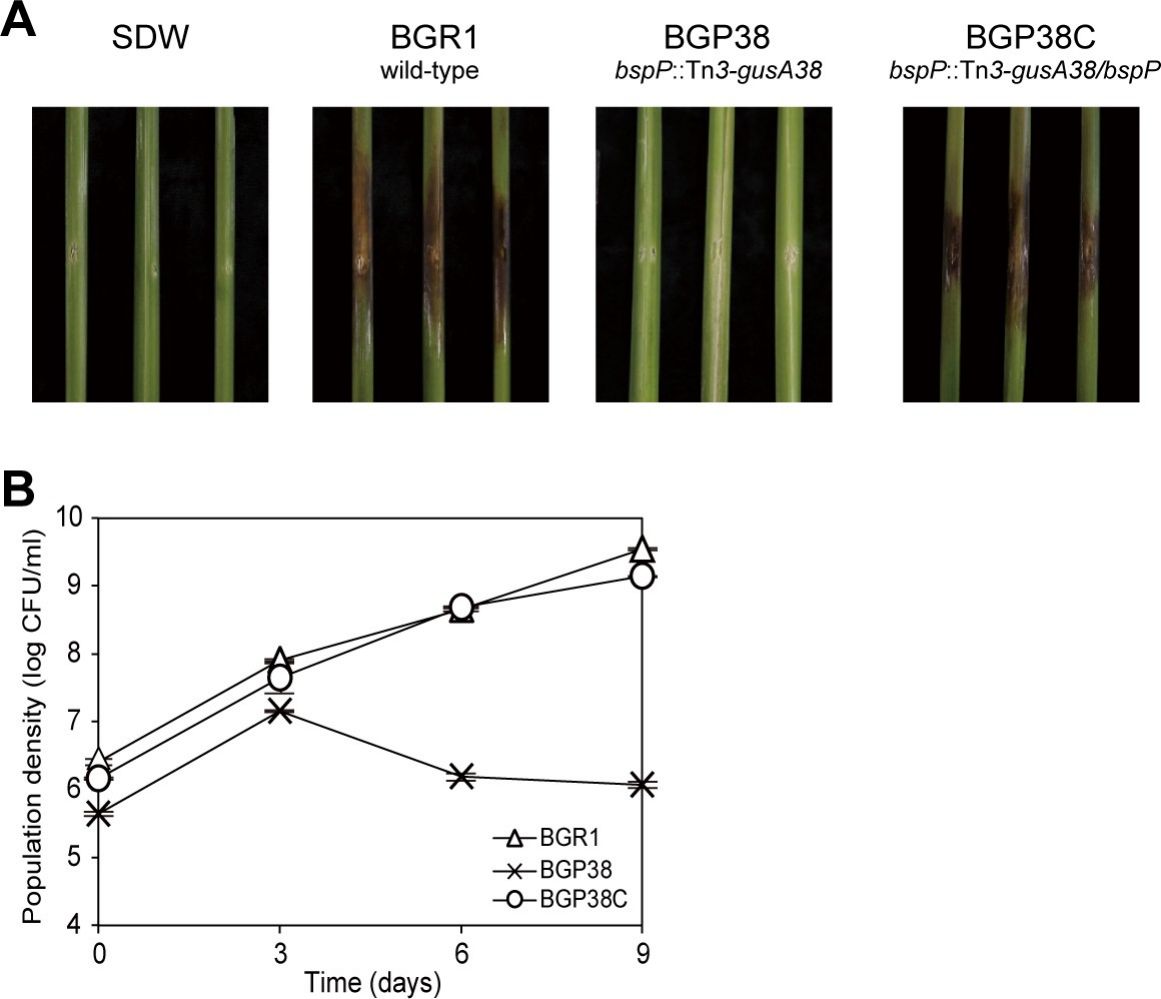
The *bspP* mutant BGP38 was avirulent and exhibited less effective colonization than wild-type BGR1. (A) The *bspP* null mutant BGP38 exhibited no visible symptoms whereas wild-type BGR1 and the complementation strain BGP38C caused severe symptoms in rice sheath. (B) The *bspP* null mutant BGP38 failed to colonize effectively in rice sheath after 9 days.

To determine whether lifestyle changes in the *bspP* null mutant BGP38 affect fitness and virulence, we inoculated rice plants with BGR1, the *bspP* null mutant BGP38, and the *bspP* complementation strain BGP38C. The ability to colonize rice plants was determined by monitoring CFUs. The *bspP* null mutant BGP38 showed no disease symptoms in the rice sheath whereas wild-type BGR1 and the *bspP* complementation strain BGP38C caused typical symptoms (Fig 7A). Wild-type BGR1 and the *bspP* complementation strain BGP38C successfully colonized the rice sheath for 9 days whereas the population of the *bspP* null mutant BGP38 decreased significantly after 3 days of incubation (Fig 7B). These results indicate that the null mutation in *bspP* reduced colonization ability in rice plants, resulting in a failure to induce disease symptoms.

## Discussion

Bacteria adapt to an ever-changing environment via genetic, physiological, and behavioral changes, a process known as bacterial environmental adaptation [30, 31]. The genetic and physiological flexibility of bacteria plays an important role in ensuring the most suitable variants are present within a niche, providing an important means of survival in undesirable environments [32, 33]. Experimental conditions such as static culture of aerobic bacteria cause limitations in available resources that pressure bacteria to adapt.

Pellicle formation of *B*. *glumae* BGR1 was the first behavioral change observed in static culture [21]. The present study demonstrated that cells of *B*. *glumae* change genetic elements to transform their physiology in a pellicle-forming microenvironment as a survival strategy. This raises important questions. First, what genetic and/or physiological changes occur at the cellular and environmental levels to increase the survival of aerobic QS bacteria under adverse growth conditions? Second, do these genetic or physiological changes affect QS itself in aerobic QS bacteria? Finally, do these genetic or physiological adaptations for improved survival increase the fitness of pathogenic bacteria in the host?

We believe our results have at least partially answered these questions. Pellicle formation is a collective behavior of *B*. *glumae* that provides a survival advantage in static culture [21]. However, the genetic and physiological natures of cells in static culture were not homogeneous. Colony variants in developed pellicles appear to tolerate stressors by forming biofilms as a survival strategy [34–37]. Similarly, we observed two types of colony variants, CS- and IR-types, which were considered the result of adaptation of individual cells in a static bacterial population. The two types of colony variants were consistently observed, and the CS-type prevailed over the IR-type. However, with the exception of colony morphology, we were unable to distinguish between the two types in terms of physiological phenotype. The IR-type was observed with the deletion mutants BGA1 and BGS8, while the R1S1 mutant, with a deletion of 321 bp near the 3’ end of *bspP*, exhibited a CS-type morphology. Because BspP has PAS-9 and SpoIIE domains that are involved in cellular signal processing, colony morphology may vary depending on the location of the mutation. Furthermore, it is unclear whether any signaling process mediated by BspP plays a role in the physiological changes observed in the *bspP* mutant.

Both types of colony variants formed facilitated pellicles compared to wild-type BGR1 and had various mutations in the *bspP* gene in *B*. *glumae*. The results of accelerated pellicle formation and c-di-GMP level measurements were in good agreement although the role of BspP in the biosynthesis or degradation of c-di-GMP is unknown. In addition to promoting pellicle formation, the environmental pH was sufficiently increased to induce toxicity; this was a result of decreased expression of *obcA* in the *bspP* mutant. We do not believe that this reduced expression of *obcA* is a function of QS, because the mutant-generated QS signals were at approximately the same level as the wild type grown in LB supplemented with HEPES. These results suggest that there may be other undiscovered factors associated with BspP that are important for *obcA* expression. Considering that BspP contains domains such as PAS-9 that act as a molecular sensor and play a key role in protein–protein interactions [38, 39], it is likely that BspP interacts with additional proteins.

Plant pathogenic bacteria that cause leaf disease multiply in apoplasts, which are rich in humidity and air and produce virulence factors [40, 41]. Thus, the infectious bacterial pathogens recognize the surrounding environment, multiply in the infection court, and finally colonize. Such colonization processes are critical for plant pathogenic bacteria to persist in hosts and produce virulence factors. Our results show that a null mutation in *bspP* conferred survival advantages in unfavorable *in vitro* growth conditions and facilitated pellicle formation. However, this change, which provides a survival advantage by sacrificing a specific gene, in this case *bspP*, was not beneficial to *B*. *glumae in vivo* as a pathogenic bacterium. These results indicate that BspP may play other roles in the pathogenic interaction between *B*. *glumae* and rice plants. Because BspP homologs have been identified in other plant pathogenic bacteria such as *B*. *gladioli*, *B*. *plantari*, and *R*. *solanacearum*, it would be interesting to determine whether BspP homologs play similar roles in these bacteria.

Because our results show experimental evolutionary aspects of a typical foliar bacterial pathogen *B*. *glumae* under artificial static culture, it is worth considering the effects of multiple passes of wild-type BGR1 in rice tissue. In addition, because *B*. *glumae* has a wide range of hosts, including rice, tomato, pepper, and potato, it will be interesting to investigate how *B*. *glumae* cells adapt to multiple rounds of inoculation and re-isolation in alternating different hosts [42]. How biofilm-producing *B*. *glumae* adapts to its host is still not known. In this study, we mainly focused on demonstrating that one of the probable functions of BspP is facilitating pellicle formation, as observed by the outcome of a specific gene (*bspP*) mutation *in vitro*. In addition to the phenotypic changes we observed *in vitro*, BspP may have other functions *in vivo*, because the *bspP* mutant was less virulent. Therefore, this case differs from the phenomenon of persistence observed in the archetypal biofilm-producing species *Pseudomonas aeruginosa* during interactions between the pathogen and lung in cystic fibrosis patients [43]. Such *in vivo* evolution experiments would improve our understanding of how cells of *B*. *glumae* evolve in nature after they encounter diverse host plants. We conclude that unidirectional evolutionary methods, such as specific genetic sacrifice, to adapt to undesirable *in vitro* growth environments are not always beneficial for the survival of plant pathogenic bacteria such as *B*. *glumae*.

## Supporting information

S1 File (PDF)
