# Supplementary material for: Adverse effects of adaptive mutation to survive static culture conditions on successful fitness of the rice pathogen Burkholderia glumae in a host
Source: PLoS One. 2020 Aug 24;15(8):e0238151. doi: 10.1371/journal.pone.0238151 (PMC7444824; doi:10.1371/journal.pone.0238151)
Supplement: S1 File — (PDF) [file pone.0238151.s001.pdf]

Adverse effects of adaptive mutation to survive static culture conditions on successful fitness of the rice pathogen *Burkholderia glumae* in host

Gi-Young Kwak[1*], Eunhye Goo[1*], Haeyoon Jeong[1], & Ingyu Hwang[1,2**]

[1]Department of Agricultural Biotechnology, Seoul National University, Seoul 08826, Republic of Korea

[2]Research Institute of Agriculture and Life Sciences, Seoul National University, Seoul 08826, Republic of Korea

[*]These authors contributed equally to this work.

[**]Corresponding author

E-mail: ingyu@snu.ac.kr

Table of Contents

S1 Fig. Autoinducer and oxalate biosynthesis and expression level of *obcA* in the *bspP* null mutant BGP38 during shaking culture of LB supplemented with 100 mM HEPES……......Page 2

S2 Fig. Phenotypic complementation of IR-type *bspP* mutant strain BGS8 to the wild-type BGR1 phenotype……………………………………………………….……….........Page 3

S1 Table. Strains and plasmids used in this study…………………………...…………Page 4-5

S2 Table. Primers used in this study ……………………………………….….…………Page 6

Supporting information references for S1Table…………………………….….…………Page 7

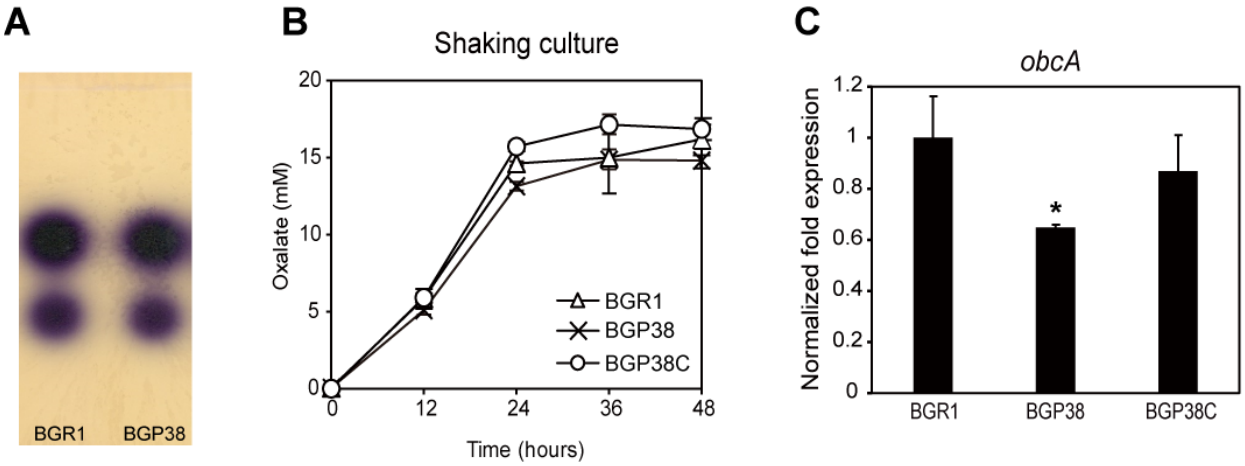

S1 Fig. Autoinducer and oxalate biosynthesis and expression level of *obcA* in the *bspP* null mutant BGP38 during shaking culture of LB supplemented with 100 mM HEPES.

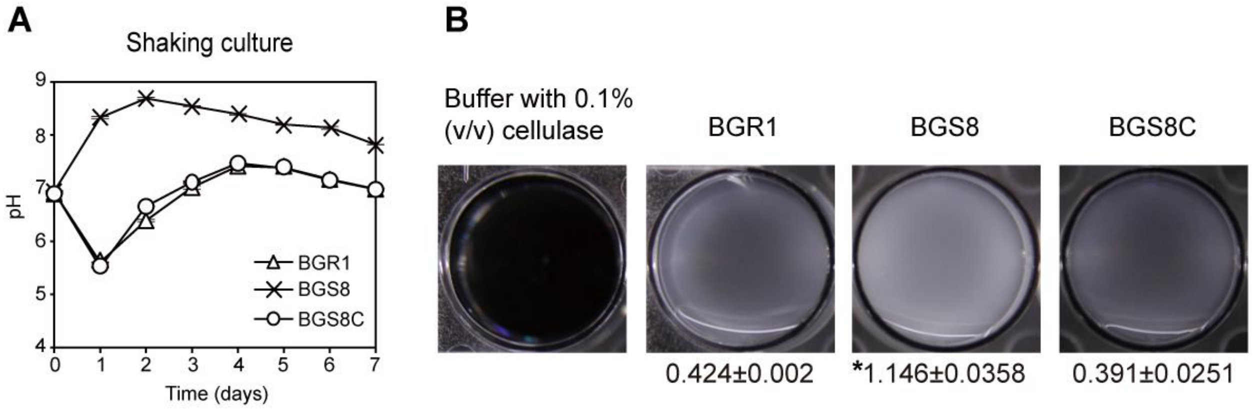

S2 Fig. Phenotypic complementation of the IR-type *bspP* mutant BGS8 with pPAS1.

S1 Table. Strains and plasmids used in this study.

Bacterial strain or plasmid	Genotype	Source or reference
Strains		
Burkholderia glumae		
BGR1	Wild-type, Rif ^{R}	[1]
3-2A	*bspP* spontaneous mutant, point mutation at 3′ end	This study
R1S1	*bspP* spontaneous mutant, deletion mutation at 3′ end	This study
R1S2	*bspP* spontaneous mutant, point mutation at 3′ end	This study
R1S3	*bspP* spontaneous mutant, deletion mutation at 3′ end	This study
R1S4	*bspP* spontaneous mutant, point mutation at 3′ end	This study
R1S10	*bspP* spontaneous mutant, deletion mutation at 3′ end	This study
		This study
BGA1	*bspP* spontaneous mutant, deletion mutation at 5′end	This study
BGA2	*bspP* spontaneous mutant, point mutation cluster at 5′ end	This study
BGS8	*bpsP* spontaneous mutant, deletion mutation at 3′ end	This study
BGS8C	BGS8, pPAS1::EZ-Tn*5*, Tp^{R}	This study
BGP38	BGR1, *bspP*::Tn*3-gusA38*	This study
BGP38C	BGP38, pPAS1::EZ-Tn5, Tp^{R}	This study
BGB107	BGR1, *bcsB*::Tn*3-gusA107*	[2]
Escherichia coli		
DH5α	F^{-} Φ80d*lacZ*Δ*M15*Δ(*lacZYA-argF*) *U169* endA1 *recA1* *hsd1hsdR17*($r_k^{-}m_k^{+}$) *deoRthi-1supE44*λ^{-} *gyrA96 relA1*	Gibco BRL
S17-1	Tra^{+}, *recA*, Sp^{R}	[3]

C2110	*polA*, Nal	[4]
Plasmids		
pBluescript II SK(+)	Cloning vehicle; phagemid, pUC derivative, Amp^R	Stratagene
pSShe	Cm^R	[4]
pHoKmGus	Promoterless *β*-glucuronidase gene; Km^R Amp^R *tnpA*	[5]
pRK2013	Tra^+, ColE1 replicon, Km^R	[6]
pLAFR3	Tra^-, Mob^+, RK2 replicon, Tet^R	[7]
pPAS1	Plasmid harboring *bspP* with Tp^R	This study

S2 Table. Primers used in this study

Oligonucleotide	Sequence (5′ → 3′)[a]	Use
BGLU_RS28885F	GTGAAACGAATCGAAGCGATCATC	*bspP* amplification
BGLU_RS28885R	TCACCGGCCAGCAGGCG	*bspP* amplification
BGLU_RS28235F	CTCGCTGCTGCTGTGCT	*bcsB* qRT-PCR
BGLU_RS28235R	AGCGTGACCAGCTGGTTGATG	*bcsB* qRT-PCR
BGLU_RS27005F	AATCGCCGGCATTATTGAA	*obcA* qRT-PCR
BGLU_RS27005R	TCGAGCGAATGGTTTCCTCG	*obcA* qRT-PCR
BSPP-1F	CGTCGACGCGTTCGTCGC	*bspP* sequence confirmation
BSPP -1R	GCAAACGACCCCGGCAGG	*bspP* sequence confirmation
BSPP 2F	GAGCTGTACTACTTCTGCACC	*bspP* sequence confirmation
BSPP -2R	CAGCAGGTCGAAGCAGCCG	*bspP* sequence confirmation
BSPP -3F	GTGCACGACYGCCTGATTGCG	*bspP* sequence confirmation
BSPP -3R	GAATTCGAACTACGCGAAGCG	*bspP* sequence confirmation
BSPP -4F	CTGACGCCCAACGGCGAC	*bspP* sequence confirmation
BSPP -4R	CGCAGGTCCGATGACGAGTC	*bspP* sequence confirmation
BSPP -5F	CTGCCGCTGCGCTTCCTGCCGTC	*bspP* sequence confirmation
BSPP -5R	GTGTACCGGTTCCAGGCGGCC	*bspP* sequence confirmation
BSPP -6F	GARCARGAACACGCCGCATGACAC	*bspP* sequence confirmation
BSPP -6R	CACCTTCGTCACCACGCATGC	*bspP* sequence confirmation

[a] Restriction sites are not shown.

Supporting information References

1. Jeong Y, Kim J, Kim S, Kang Y, Nagamatsu T, Hwang I. Toxoflavin produced by *Burkholderia glumae* causing rice grain rot is responsible for inducing bacterial wilt in many field crops. Plant Dis, 2003;87: 890–895.
2. Kwak G-Y, Choi O, Goo E, Kang Y, Kim J, Hwang I. Quorum sensing-independent cellulase-sensitive pellicle formation is critical for colonization of *Burkholderia glumae* in rice plants. Front Microbiol. 2020;10: 3090. Available from: https://www.frontiersin.org/articles/10.3389/fmicb.2019.03090/full
3. Simon R, Priefer U, and Pühler A. A broad host range mobilization system for *in vivo* genetic engineering: Transposon mutagenesis in gram-negative bacteria. Nat Biotechnol. 1983;1: 784–791.
4. Stachel SE, An G, Flores C, Nester EW. A Tn*3lacZ* transposon for the random generation of beta-galactosidase gene fusions: Application to the analysis of gene expression in *Agrobacterium*. EMBO J. 1985;4: 891–898.
5. Bonas U, Stall RE, Staskawicz B. Genetic and structural characterization of the avirulence gene *AvrBs3* from *Xanthomonas campestris* pv. *vesicatoria*. Mol Gen Genet. 1989;218: 127–136. Available from: https://doi.org/10.1007/BF00330575
6. Figurski DH, Helinski DR. Replication of an origin-containing derivative of plasmid RK2 dependent on a plasmid function provided in *trans*. Proc Natl Acad Sci U S A. 1979;76: 1648–1652.
7. Staskawicz B, Dahlbeck D, Keen N, Napoli C. Molecular characterization of cloned avirulence genes from race 0 and race 1 of *Pseudomonas syringae* pv. *glycinea*. J Bacteriol. 1987;169: 5789–5794.
